# Spatial-Temporal Patterns of Viral Amplification and Interference Initiated by a Single Infected Cell

**DOI:** 10.1128/JVI.00807-16

**Published:** 2016-07-27

**Authors:** Fulya Akpinar, Bahar Inankur, John Yin

**Affiliations:** Systems Biology Theme, Wisconsin Institute for Discovery, Department of Chemical and Biological Engineering, University of Wisconsin—Madison, Madison, Wisconsin, USA; Wake Forest University

## Abstract

When viruses infect their host cells, they can make defective virus-like particles along with intact virus. Cells coinfected with virus and defective particles often exhibit interference with virus growth caused by the competition for resources by defective genomes. Recent reports of the coexistence and cotransmission of such defective interfering particles (DIPs) *in vivo*, across epidemiological length and time scales, suggest a role in viral pathogenesis, but it is not known how DIPs impact infection spread, even under controlled culture conditions. Using fluorescence microscopy, we quantified coinfections of vesicular stomatitis virus (VSV) expressing a fluorescent reporter protein and its DIPs on BHK-21 host cell monolayers. We found that viral gene expression was more delayed, infections spread more slowly, and patterns of spread became more “patchy” with higher DIP inputs to the initial cell. To examine how infection spread might depend on the behavior of the initial coinfected cell, we built a computational model, adapting a cellular automaton (CA) approach to incorporate kinetic data on virus growth for the first time. Specifically, changes in observed patterns of infection spread could be directly linked to previous high-throughput single-cell measures of virus-DIP coinfection. The CA model also provided testable hypotheses on the spatial-temporal distribution of the DIPs, which remain governed by their predator-prey interaction. More generally, this work offers a data-driven computational modeling approach for better understanding of how single infected cells impact the multiround spread of virus infections across cell populations.

**IMPORTANCE** Defective interfering particles (DIPs) compete with intact virus, depleting host cell resources that are essential for virus growth and infection spread. However, it is not known how such competition, strong or weak, ultimately affects the way in which infections spread and cause disease. In this study, we address this unmet need by developing an integrated experimental-computational approach, which sheds new light on how infections spread. We anticipate that our approach will also be useful in the development of DIPs as therapeutic agents to manage the spread of viral infections.

## INTRODUCTION

Viruses cause diseases by interacting with their hosts across multiple length scales. Interactions within a single cell between an invading viral genome and the host cell resources lead to the expression of viral genes, replication of progeny genomes, and assembly of viral progeny particles. Upon release from their host cell, progeny particles are transported by diffusion or convection to nearby or distant susceptible cells and initiate new rounds of infection, often triggering or engaging with innate cellular immune responses or systemic adaptive immune responses that influence the progression of disease in a complex living host. While the study of the interactions at each scale can provide valuable data about virus infections, it is becoming increasingly clear that a comprehensive picture of virus infections that accounts for and incorporates virus-host interactions across multiple length scales (from the molecular to the cell and tissue levels, as well as the systemic level) and across multiple time scales (from minutes to hours to days) will be needed to frame in an appropriate context the major challenges facing molecular and cell scientists, clinical virologists, and epidemiologists (1–3). Moreover, such perspectives may enable the identification and development of new approaches toward the design of antiviral strategies, particularly strategies for preventing viral escape (4–7).

A majority of the existing multiscale studies linking intracellular virus growth to cell-to-cell infection spread features are based on computational models that describe basic interactions between replicating viruses and immune responses (3, 8, 9). However, the differential equations that define such models assume well-mixed conditions and do not account for the inevitable spatial heterogeneities that contribute to within- and between-host dynamics in natural infections. Spatial variation can be incorporated by recognizing that virus particle movement by physical processes such as diffusion or fluid flow may combine with biological processes of particle amplification, leading one to write and solve sets of coupled partial differential equations (PDE) or population balance models (3, 10, 11), which can be challenging to implement. Hence, simpler agent-based cellular automaton (CA) models that enable the reproduction of the discrete nature of biological systems and link intracellular and extracellular components of virus infections have been developed (12–16). However, CA models generally have tenuous links to experiments; their model parameters have little connection to molecular or cellular wet-lab measurements. Moreover, single cells exhibit extremely heterogeneous behavior associated with the production of virus progeny (17–21), which likely influences how infections spread. However, such behaviors have yet to inform PDE or CA models of infection spread.

To date, multiscale studies of infections have focused on the dynamics of viable virus particles while avoiding consideration of the plethora of nonviable or defective particles that accompany the production of most viable particles (22–24). Noteworthy are defective interfering particles (DIPs), virus-like particles that carry genomes lacking one or more viral genes that are essential for growth. When viable virus and their DIPs coinfect the same host cell, the defective genomes compete with the intact genomes for viral proteins, resulting in a reduction and delay in infectious virus production. Further, DIPs activate host immune responses both *in vitro* (25–30) and *in vivo* (31–33), motivating the use of natural or engineered DIPs in antiviral formulation studies (33–35). DIPs not only influence virus infections when administered externally but have also been detected during natural infections of human hosts by influenza A virus (36, 37) and dengue virus (38, 39), as well as in avian hosts infected by West Nile virus (40). The presence of DIPs in multiple virus populations in nature and recent findings suggesting the cotransmissibility of DIPs among individuals (37, 39) indicate the potential impact of DIPs on the multiscale progression of acute *in vivo* infections.

Coinfections of host cells with DIPs and their viable intact viruses have provided evidence that DIPs inhibit the synthesis of viral genomes, protein, and infectious progeny virions (41–46). Further, we have recently elucidated the effects of the DIP dose at the single-cell level, quantifying both the extent and the extreme variability of the interfering effects of DIPs on intracellular viral gene expression and viable particle production (47). However, little is known about the effects of DIPs on virus spread. Theoretical models, in the absence of experimental observations or parameters, suggest that infections can fluctuate or persist (48). In the only experimental study of the impact of DIPs on infection spread, Clark et al. (49) observed that the addition of DIPs leads to a delay in infection spread *in vitro*, but they did not characterize or quantify the spread features.

Here we consider the spread of a recombinant vesicular stomatitis virus (VSV) that has been engineered to express red fluorescent protein (RFP) (50). We monitor its plaque growth on cell monolayers in the presence of DIPs, and we observe a diversity of dose-dependent infection spread patterns. These observations provide the first experimental evidence for long-range temporal and spatial effects of DIPs in their intracellular interference with virus growth kinetics. Moreover, to extend our experimental observations, we combine them with CA modeling and examine the impact of extracellular processes (i.e., diffusion and virus adsorption) on predicted patterns of coinfection spread. The model incorporates our experimentally determined correlations between the DIP multiplicity and the distributions of kinetic parameters of infection measured at the single-cell level (47). This work is the first data-driven model to show that stochastic gene expression at the single-cell level can amplify and propagate over multiple cycles of virus growth and infection spread. Moreover, by explicitly accounting for DIPs, the model enables us to simulate spatial patterns of both virus and DIP spread and to explore diverse coinfection scenarios, including those needed for full inhibition of the spread of virus infection. Overall, this work sets the foundation for predictive multiscale models for other virus-host systems. Finally, insights from this approach will advance our understanding and application of natural or engineered virus infection spread in the presence of DIPs.

## MATERIALS AND METHODS

### Cell type and virus strain.

Baby hamster kidney cells (BHK-21 cells) were grown in minimal essential medium (MEM; Corning) with 10% fetal bovine serum (FBS; Atlanta Biologicals) and 2 mM GlutaMAX I (Gibco). The cell line was cultured in a humidified incubator at 37°C under 5% CO_2_. The infections were carried out using a recombinant vesicular stomatitis virus strain (VSV-rWT-DsRed-Ex) engineered to carry and express a DsRed-Express-DR gene (Clontech) (RFP) as a by-product of infection (50). DIPs were produced by fixed-multiplicity serial infections using VSV-rWT-DsRed-Ex at a multiplicity of infection (MOI) of 10, and the passage with the highest DIP concentration (determined by a yield reduction assay) (45) was stored at −80°C for later infections (47). No RFP expression was observed in cells infected with purified DIPs, reflecting a loss of functional RFP during DIP generation; such DIPs, generated by high-MOI passage, may well exist as a mixture of DIPs carrying genomes of different lengths and exhibiting different extents of interference (51).

### Solution-phase coinfection and plating of cells.

Coinfections were carried out in solution as described previously (18, 47). Monolayers of BHK-21 cells grown in T75 cell culture flasks (BD Falcon) were released by treatment with trypsin (Cellgro) after washing with Dulbecco's phosphate-buffered saline (DPBS; Gibco). The cell suspension was diluted to 10^5^ cells/ml in MEM, cooled on ice, and mixed with VSV-rWT-DsRed-Ex (MOI, 30) and with DIP (multiplicity of DIP [MODIP], 10, 1, 0.1, or 0) in the cold for 30 min, to minimize cell aggregation and allow for virus attachment without entry; a high MOI (MOI, 30 to 50) ensures that virtually all cells, even resistant ones, become productively infected with viable virus (50). The temperature of the virus-cell solutions was raised to 37°C in a water bath for 5 min to allow for internalization of the attached virus and DIPs. The infected-cell suspension was then centrifuged at 1,000 rpm for 4 min at 4°C, the medium was discarded, and the pellet was resuspended. This procedure was repeated three times to minimize the carryover of unbound virus and DIPs from the infected-cell suspension, which was then diluted to a final concentration of 30 infected cells/ml in a suspension of 5 × 10^5^ noninfected cells/ml. The solution containing infected and noninfected cells was added to 12-well plates (1 ml/well). The cells were allowed to attach to the plate for 1 h, and the medium was then replaced with an overlay of semisolid 2% FBS in MEM containing 0.6% (wt/vol) agar. Infection was allowed to proceed in a humidified incubator at 37°C under 5% CO_2_.

### Live-cell time lapse imaging.

Time lapse microscopy of plaque formation was performed using a Nikon Eclipse Ti microscope with a QICAM Fast 1394 digital camera (QImaging). The plate was placed in a stage-top incubation chamber at 37°C, 5% CO_2_, and 85% relative humidity, and the temperature was maintained by an outer warming chamber encompassing the microscope (InVivo Scientific). Starting at 3 h postinfection, each well was imaged at a magnification of ×4, capturing ∼75% of the well area every 2 h for 28 h, after which the single plaques started to merge under low-MODIP conditions. Only the plaques initiated with a MODIP of 10 (highly inhibited) could be imaged for as long as 37 h. At the end of the experiment, the plates were scanned with a GE Typhoon FLA 9000 biomolecular imager (555/580 nm) to obtain the total plaque counts for each well. Overall, 17, 8, 24, or 48 plaques were obtained and examined in the wells with cells infected at a MODIP of 0, 0.1, 1, or 10, respectively. By normalizing the number of emerged plaques to the number of plated infected cells, the fraction of fully inhibited spread was estimated for each MODIP condition. Imaging through the course of infection did not affect the size of the plaques, as revealed by comparison of the areas of imaged and nonimaged VSV-rWT-DsRed-Ex plaques in the absence of DIPs (data not shown).

Prior to the initiation of each time lapse run, images were taken to correct for uneven light intensities. A red fluorescence reference slide (Ted Pella, Inc.) was used for illumination and dark-field correction images.

### Image processing and quantification.

The initial batch image organization and processing were done using Je'Xperiment (JEX-0.0.1; source code available at http://sourceforge.net/projects/jextools), a Java-based image-processing and quantification interface (20). Briefly, the images were first sorted according to the experimental condition, location in the array, and time. After correction of the image background using calibration images, individual plaques in corrected images were cropped and were analyzed using custom-written macros in Fiji (52). The locations of plaque centers (P.C.) were determined automatically based on early time images of the plaques using detection of maxima. The area of each plaque was quantified at each time point based on the fluorescence-positive area. The area equivalent radius (AER) of the measured area was calculated by determining the radius of the circle that would have the same area as the measured area. Plaques were categorized according to their expansion rates. Plaques growing at a spread rate that was within the measured range of plaques initiated at a MODIP of 0 were counted as normal plaques. On the other hand, plaques growing at a spread rate below this range were considered slow-growing or patchy plaques, depending on their spatial characteristics. The determination of further measures, including the percentage of the fluorescence-positive area and the mean fluorescence intensity over the area of each plaque, is presented below.

### Concentric ring quantification of plaque characteristics.

**FIG 1:**
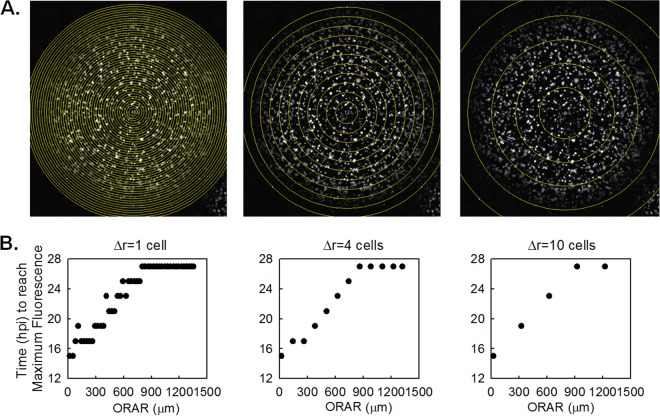
Determination of concentric ring radii. (A) Concentric rings with increments in radial distance (Δr) of 1, 4, and 10 cell diameters (30 μm/cell) are shown with a maximum-intensity z-stack projection of the time course images of a normal plaque. The outer radii of analyzed rings (ORAR) are marked as yellow circles. (B) The time at which the maximum mean RFP fluorescence is reached (i.e., one round of infection is completed) at each ring (ordinate) is plotted with respect to the ORAR (abscissa). When Δr is 1 cell, the RFP intensities in multiple adjacent rings reach the maximum level at the same time, indicating that the infections occur with close timing in the cells of the adjacent rings. On the other hand, increasing Δr to 10 cells masks the complete infection events that could be tracked using a Δr of 4 cells. In our selected case of a Δr of 4 cells, each ring represents a separate single round of infection spread.

Once the spatial locations of plaque centers were defined, the time point images were stacked to create the maximum-intensity Z-projections of each plaque, on which concentric circles around the plaque center with radial interval distances of approximately 4 cell diameters (30 μm/cell) were drawn to form rings representing the rounds of infection spread (Fig. 1). After the background for each image was set at zero, the percentage of the area that was positive for fluorescence and the mean fluorescence intensity on each ring were extracted using Fiji, and the measurement data tables were analyzed using MATLAB, release 2012a (MathWorks, Natick, MA). The mean intensity values were normalized to the average mean intensity at the centers of plaques formed in the absence of DIPs (at a MODIP of 0). The data extracted from each ring were plotted with respect to the outer radius of the ring.

### Quantification of spatial and temporal features of spread.

Temporal features of spread, including the spread rate, delay, and fold decrease in spread rate, were extracted from plaque expansion (area equivalent radius over time) profiles using MATLAB. Spread rate was calculated as the slope of linear approximation of plaque expansion profiles, while delay in spread was the interval between the time of virus adsorption and the time when fluorescence expression at the plaque center first became detectable. Because the spread rates of some plaques were not linear, the fold decrease in the spread rate was estimated as the ratio of the average spread rate for the final three time points to the average postdelay spread rate for the earliest three time points. Spatial features of spread were obtained from viral activity profiles over concentric rings. First, the normalized percentage of the area that was fluorescence positive and the mean fluorescence intensity of this area in the 4-cell-diameter circle around the plaque center were quantified. Second, the reductions in these two viral activity measures within the 8-cell-diameter distance were calculated.

### Cell automaton model.

The cell automaton model was developed using MATLAB 2012a, and as described in Results, the model was guided by experimentally determined intracellular probabilistic rules (47) and equations 1 to 6, governing extracellular events. The simulation of each plaque was run with a time step of 6 min, typically over a full duration of 30 to 70 h. Distributions of spread features for each condition were extracted from 200 simulation runs. The parameters of the model were optimized by minimizing the least-square error between measured and modeled spread features, summed over all MODIP conditions. Optimization was performed to match either the spread rate or spatial features, such as the normalized percentage of the area that was fluorescence positive and the relative mean fluorescence intensity around the plaque center, and their decrease. Since spatial features were multiple, the least-square errors of all spatial features were summed.

The sensitivities of the model parameters were calculated by comparing the optimum model results to the distributions generated after perturbing one parameter in each optimum parameter set, while fixing all other parameters at their optimum values. The adsorption efficiency, diffusion constant, and clearance rate were perturbed by 0.1, and the superinfection period was perturbed by 0.5 h. For comparison, the Mann-Whitney U test was used, and *P* values were evaluated to score the significance of change. A *P* value of <0.01 was assumed to be a statistically significant change.

## RESULTS

### Spread patterns in the presence and absence of DIPs.

To investigate the effect of DIPs on infection spread, we tracked infectious virus propagation on BHK-21 cell monolayers using a recombinant vesicular stomatitis virus (VSV) strain expressing red fluorescent protein (RFP). RFP provides a near-real-time report of viral gene expression, correlating with the timing of viral progeny release from infected cells, and is also a useful tool for probing the effects of DIPs on viral activity at the single-cell level (47). To avoid potentially confounding the immune activation functions of DIPs, we used BHK-21 cells, which exhibit minimal antiviral activity (53, 54). Each well contained at most 30 infected or coinfected cells along with a large population of healthy cells. The spatial propagation of infection was tracked by fluorescence microscopy for as long as 37 h postinfection (hpi) using conditions set to minimize cell death due to phototoxicity or cell aging.

**FIG 2:**
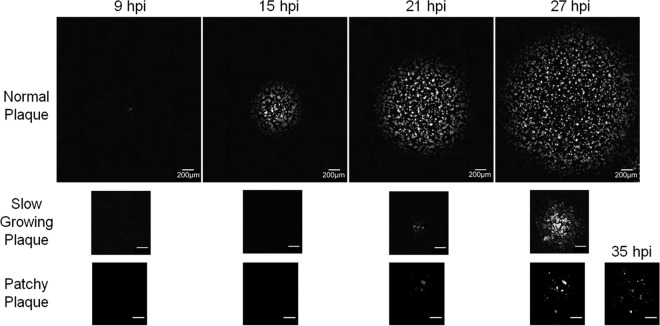
Spread patterns in the presence and absence of DIPs. Representative time lapse images of three major spread patterns on BHK-21 cells infected with reporter VSV at an MOI of 30 and their DIPs at various multiplicities are shown. Bars, 200 μm. Normal plaques (top) emerged from cells infected at all MODIP levels, but primarily at a MODIP of 0 or a low MODIP (0.1 or 1). Slow-growing (center) and patchy (bottom) plaques were observed only in the presence of DIPs (MODIP levels, 1 and 10). Time points are shown above the panels. Since the patchy plaques developed more slowly than the others, an additional image at 35 hpi is shown. See also Movies S1 to S3 in the supplemental material.

Time lapse imaging of plaque formation at different MODIP levels revealed three patterns of virus spread: normal, slow growing, and patchy (Fig. 2). Normal plaques expanded symmetrically and homogeneously with the initial infection and became visible around 9 hpi. Similarly, slow-growing plaques were symmetric and homogeneous, but their initial appearance was delayed relative to that of normal plaques. In contrast, patchy plaques appeared after still longer delays and exhibited highly irregular shapes.

### Patterns of infection spread depend on the initial DIP dose.

**FIG 3:**
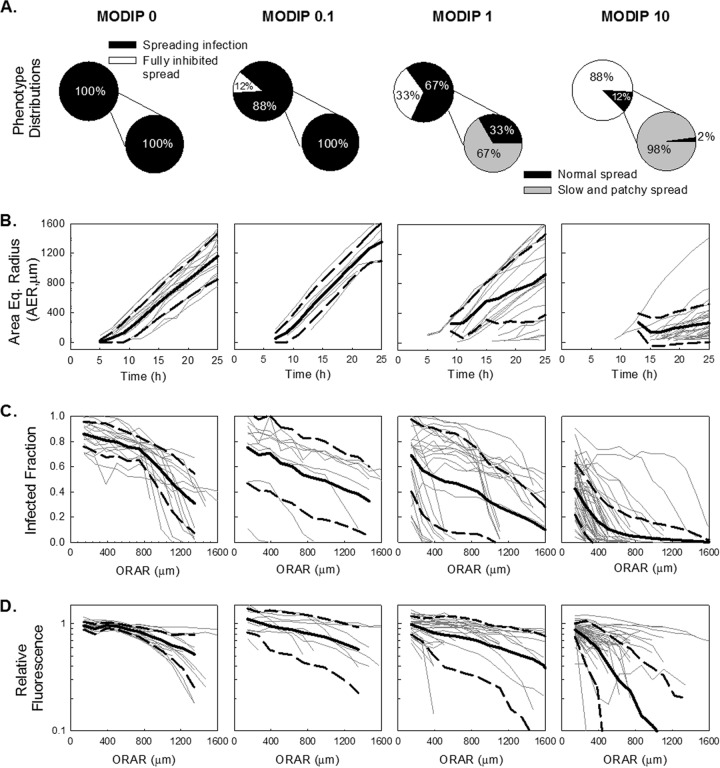
Quantitative analysis of infection spread. Features extracted from spread patterns initiated by single cells coinfected with different DIP levels (given at the top) are shown. A total of 17, 8, 24, and 48 plaques were imaged in three experimental trials at MODIP levels of 0, 0.1, 1, and 10, respectively. (A) Spread phenotype distributions are shown in pie charts. Upper pie charts show the percentages of spreading infections (black) and fully inhibited spread (white), while lower pie charts show the percentages of normal spread (black) and slow/patchy spread (gray) as subsets of the spreading infections. (B) The expansion profiles of each plaque at each MODIP are shown as gray lines; the means and standard deviations of these profiles are shown as solid and dashed black lines, respectively. The ordinate is the area equivalent radius (AER), calculated from the circle that has the same area as the plaque, and the abscissa is time. (C) Infected fraction (ordinate) calculated based on the fraction of the fluorescence-positive area in concentric rings around the initially infected plaques with different outer radii of analyzed rings (ORAR) (abscissa). As in panel B, individual plaque trajectories are indicated by gray lines, while means and standard deviations are shown as solid and dashed black lines, respectively. (D) The relative fluorescence in the fluorescence-positive area (ordinate) is plotted against the ORAR (abscissa). Relative fluorescence is calculated by normalizing mean fluorescence readouts to the mean fluorescence at the plaque center at a MODIP of 0.

Analysis of infection spread initiated from single cells coinfected with virus and DIPs showed a monotonic relationship between the MODIP of the initially infected cell and phenotype distributions (Fig. 3A). As more DIPs were added in the initial infection of cells, fewer cells were able to produce sufficient viral progeny to trigger the infection of neighboring cells (Fig. 3A, upper pie charts). At a MODIP of 10, only 12% of initially infected cells were able to initiate spreading infections, and only 2% of the resulting plaques expanded normally. Moreover, slow-growing and patchy plaques were not observed in the absence of DIPs, but they outnumbered normal plaques for coinfections at a MODIP of 1 or 10 (Fig. 3A, lower pie charts).

We further analyzed the infection spread patterns of each plaque for expansion profile and viral activity. By use of the florescence signal as an indicator of the progress of infection, the area covered by infected cells was quantified during the course of the experiment for each plaque at different DIP levels, and a circle with the same area was drawn; the radius of that circle was defined as the area equivalent radius (AER) (Fig. 3B). In the absence of DIPs (MODIP, 0), all plaque infections spread with a constant radial velocity, as observed previously (55, 56). Increasing the MODIP led to a decrease in the average plaque expansion rate (Fig. 3B) and an increase in the variability of expansion profiles (Fig. 3B, dashed black lines), especially at a MODIP of 1 or 10.

To quantify viral activity during infection spread, we measured the mean fluorescence intensity averaged over the concentric rings centered on the initially infected cell (plaque center). Based on these measurements, we determined the fraction of cells with detectable viral activity (producer cells) within each concentric ring (Fig. 3C). We also determined the fluorescence intensity in these infected cells in each concentric ring, normalized to the fluorescence at the P.C. in the absence of interference, or a MODIP of 0 (Fig. 3D). At a MODIP of 0, the infected fraction and the relative fluorescence remained relatively constant over the course of 28 hpi, up to a radius of 800 μm (Fig. 3C and D, leftmost graphs). Beyond this distance, both measures decreased, indicating the earliest stages of infection initiation at the largest radii, corresponding to the dynamic leading edge of the infection front (57). As the MODIP of the initially infected cell increased, the active infection front was confined to an area closer to the plaque center, and viral activity was notably reduced (Fig. 3C and D), owing to a lower rate of spread and greater patchiness of the plaques. These changes accompanied an increase in variability that peaked at a MODIP of 1 (Fig. 3C and D, dashed lines). We did not observe any further increase in variability at a MODIP of 10, potentially reflecting the exclusion of the fully inhibited spread patterns, which were highest at this MODIP.

### The initial DIP level reduces the rate of infection spread.

**FIG 4:**
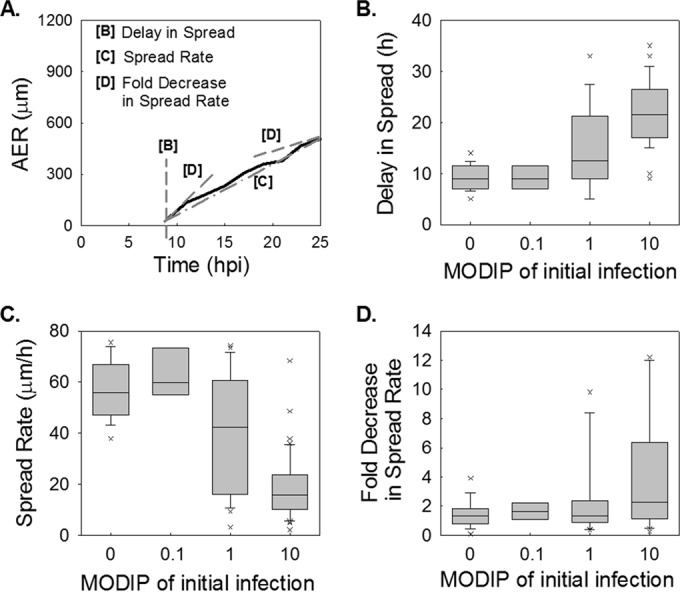
Interfering effects of initial DIPs on initial delay and the subsequent rate of infection spread. (A) Schematic illustration of how parameters are extracted from distance-versus-time data (solid line). Delay in spread, spread rate, and fold decrease in spread rate were extracted from plaque expansion profiles, shown as the area equivalent radius (AER) versus time. (B to D) The distributions of extracted features at each MODIP (abscissa) are shown as box plots. The gray boxes cover 25%-to-75% confidence intervals, while whiskers span 5% to 95%. The horizontal lines in the boxes represent medians, and each outlier is indicated by a lowercase letter x. (B) Delay is the first time point at which the fluorescence intensity exceeds the lower detection limit in the initially infected cell. (C) The spread rate is calculated by linear approximation of expansion profiles. (D) The fold decrease in the spread rate is the ratio of the instantaneous spread rate (slope of line [D] in panel A) at the last three time points to that at the earliest three time points.

The delay in spread initiation, defined as the time elapsed from virus adsorption to the initial detection of RFP expression at the plaque center, could be estimated from AER-versus-time plots for each plaque (Fig. 4A). In the absence of DIPs, the shortest delays were 6 hpi (Fig. 4B), in agreement with previously observed virus production and RFP maturation rates (50). At higher initial DIP doses, delays increased to a maximum of 35 hpi (at a MODIP of 10) (Fig. 4B). After the initial delay, the rates of infection spread during subsequent infection cycles also reflected the DIP dose of the initial cell. Specifically, spread rates of 55 μm/h in the absence of DIPs dropped to ∼15 μm/h when the cell was initially coinfected at a MODIP of 10 (Fig. 4C). Moreover, the relatively constant spread rates in the absence of DIPs during the expansion of individual plaques gave way to changing (decreasing) spread rates at higher initial DIP levels (Fig. 4D).

### DIPs reduce the level of viral gene expression and the probability of plaque formation.

**FIG 5:**
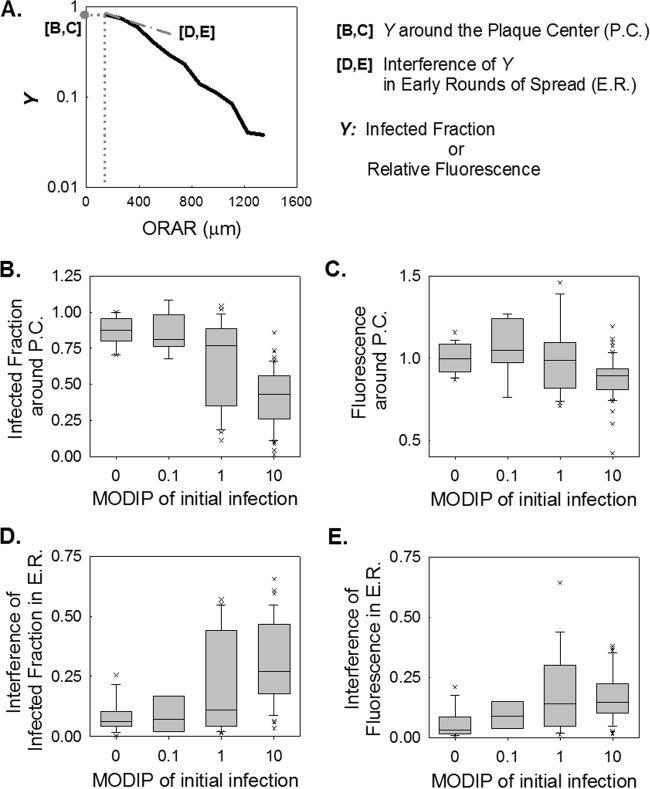
Interfering effects of DIPs on viral activity during infection spread. (A) Schematic illustration of how parameters are extracted from data depicting the infection measure (*Y*) versus the outer radius of each analyzed ring (ORAR). (B to E) Distributions of extracted features at each MODIP (abscissa) are shown as box plots. The gray boxes cover 25%-to-75% confidence intervals, while the whiskers span 5% to 95%. The horizontal lines in the boxes represent medians, and each outlier is indicated by a lowercase letter x. The infected fraction (B) and relative fluorescence (C) around the plaque center (P.C.), as well as the interference with these values within ∼8 cell diameters in early rounds of spread (E.R.) (D and E), were extracted from the infected fraction and relative fluorescence over the ORAR trajectories of the individual plaques shown in Fig. 3C and D.

Since the intensity of reporter fluorescence reflects the level of viral gene expression (47), we examined in greater depth the fluorescence intensity profiles shown in Fig. 3C and D. First, we quantified the fraction of successful infections and the viral activity levels in the proximity of the initially infected cell (within 4 cell diameters of the plaque center), as shown in Fig. 5A to C. When the initial infection was initiated at a low MODIP, ∼90% of the cells in this center region were reporter positive, corresponding to virus production (Fig. 5B). However, at high DIP levels, the fraction of reporter-positive cells decreased (Fig. 5B and C), and at a MODIP of 10, only ∼50% of cells were reporter positive, and the maximum level of gene expression was less than 80% of the level in normal (no-DIP) infections.

We further calculated the drop in successful infections and gene expression within 8 cell diameters of the initially infected cell (Fig. 5D and E), which would be directly affected by the virus released from the initially infected cell, and plausibly more under the influence of the initial DIP level than later, more spatially segregated rounds of infection. In the absence of DIPs, the drop in viral reporter activity was negligible, but it became significant with increasing DIP levels (Fig. 5E). Similarly, the fraction of successful infections fell by as much as ∼0.3 (MODIP, 10) near the plaque center, providing evidence of DIP production, release, coinfection, and escalation of interference during infection spread (Fig. 5D).

### The CA model incorporates biological and physical contributions to infection spread.

**FIG 6:**
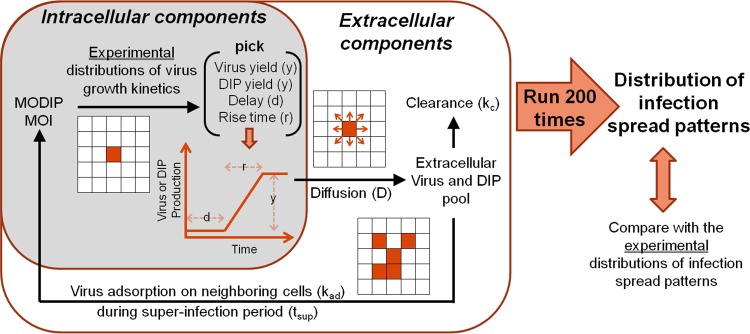
Data-driven cellular automaton model of virus-DIP interactions during infection spread. Infection starts at the center of a square grid at a given initial MODIP and MOI. As described in the shaded box, the experimentally determined mathematical relationships linking the MODIP to distributions of virus growth kinetics (virus yield, delay, and rise time) (47) are used to generate virus growth profiles. DIP yields from single cells are calculated as the difference between the infectious virus yield and maximum virus production from a cell. Virus growth is assumed to be linear and continuous. Once the virus and DIPs are released to the extracellular pool at a specific cell location, they can diffuse to neighboring cells at a particular diffusion constant (*D*), adsorb onto uninfected cells at a certain adsorption efficiency (*k*_ad_) during the superinfection period (*t*_sup_) of cells, or clear at a particular clearance rate (*k_c_*). The model runs for the desired period (e.g., 30 hpi) with time steps of 6 min, satisfying forward Euler stability criteria. By 200 iterations of the model, a distribution of infection spread patterns at a given set of model parameters and a given initial MODIP can be obtained and compared with experimental results.

When virus and DIPs coinfect a cell, the cell produces virus and DIP progeny, which may then move by diffusion to nearby cells and initiate new rounds of coinfection, ultimately generating patterns of infection spread in space and time. To examine how these processes together contribute to the pattern of infection spread, we developed a cellular automaton (CA) model (Fig. 6).

In the model, the cell monolayer is represented as a square grid in which each square is occupied by a cell (diameter, 30 μm). In this way, we represent the uniformly high cell density that we observe in experiments. Cell proliferation, death, and aging are assumed to be negligible, since no significant effect of these phenomena was observed within the relatively short duration of our experiments. Also, antiviral host responses are not included in the model, reflecting the lack of an intact immune response in BHK-21 cells (53, 54).

Initially, all the cells on the grid are healthy and susceptible to infection, except for the center cell, where the initial infection starts at a given MOI and MODIP, leading to the local production of virus and DIPs. The production profiles are generated approximating linear and continuous growth, with inputs of latent time, yield, and rise time picked randomly from previously measured distributions of these parameters (47). Although RFP expression is an intracellular measure, its correlation with infectious virus production is used to estimate the release of extracellular infectious virus. Further, DIP yields from single cells are calculated as the difference between the maximum virus production from a cell (no DIPs) and the reduced infectious virus yield owing to DIP coinfection, assuming a conservation of total particle production in DIP coinfections (51). In short, an increase in DIP production corresponds with a decrease in infectious virus yield.

As the experimental distributions of virus growth kinetics set the intracellular probabilistic rules of our model, extracellular processes determine the propagation of the viruses and DIPs produced to neighboring cells. These extracellular processes, described by equations 1 to 6, include (i) diffusion, (ii) adsorption of virus to the cell surface, and (iii) virus clearance (Fig. 6). The terms in equations 1 to 6 are defined in Table 1.

(1) $$V*(x_{i},t)=V\left(x_{i},t-dt\right)+\text{Virus generation}+\text{Diffusion}$$

(2) $$V\left(x_{i},t\right)=V*(x_{i},t)-\text{Clearance}-\text{Adsorption}$$

(3) $$\text{Diffusion}=D\cdot \underset{x_{n}}{\sum }\frac{\left[V(x_{n},t-dt)-V\left(x_{i},t-dt\right)\right]}{n_{c}}$$

(4) $$\text{Virus generation}=\overset{t}{\underset{t-dt}{\sum }}\frac{\text{Yield}\left(x_{i},t-dt\right)}{n_{c}}$$

(5) $$\text{Clearance}=k_{c}\cdot V*\left(x_{i},t\right)$$

(6) $$\text{Adsorption}={\left[(1-k_{c})\cdot V*\left(x_{i},t\right)\right]}^{k_{\text{ad}}}$$

**TABLE 1:** Variables and parameters of the model^*a*^

Parameter	Definition
*x_i_*	Current cell position
*x_n_*	Neighboring cell position
*t*	Time
*dt*	Time step
*D*	Diffusion coefficient
*n_c_*	No. of neighboring cells
*k_c_*	Clearance rate
*k*_ad_	Adsorption efficiency
*V*	Extracellular virus or DIPs

a V*, extracellular virus or DIPs; V, intracellular virus or DIPs.

### (i) Diffusion.

Virus particles and DIPs are released from each cell, creating extracellular pools of virus and DIP associated with the infected-cell location (equations 1 and 4), and these particles diffuse to the nearest four orthogonally neighboring cells, a pattern known as the von Neumann configuration. Diffusion is governed by an estimate of the concentration gradient and the diffusion constant (*D*) (equation 3), which is assumed to be less than 10 μm^2^/s, in accordance with previous measures (15).

### (ii) Virus adsorption to susceptible cell surfaces.

If extracellular virus and DIPs colocalize with an uninfected cell, they can adsorb to receptors on the cell surface. Experimentally, virus adsorption is not fully efficient, so only a fraction of virus and DIPs are internalized into a cell (58). The extent of virus adsorption is described by equation 6, in which *k*_ad_, the efficiency of virus adsorption, is less than 1 and is assumed to be the same for both infectious virus and DIP, reflecting their similar cell entry behaviors (59). Virus adsorption onto the same cell can continue up to ∼3 h, a superinfection period (*t*_sup_), following initial virus exposure, based on previous observations (60). In the model, MOI and MODIP values are calculated based on the accumulated extracellular virus and DIPs during this superinfection period.

### (iii) Virus clearance.

A fraction of extracellular viruses and DIPs is assumed to be cleared at each time step (calculated by use of the clearance rate [*k_c_*] in equation 5), to account for the loss of virus due to degradation or potential immobilization of virus particles and DIPs in the agar overlay.

### The CA model replicates observed DIP-mediated reduction of infection spread.

**FIG 7:**
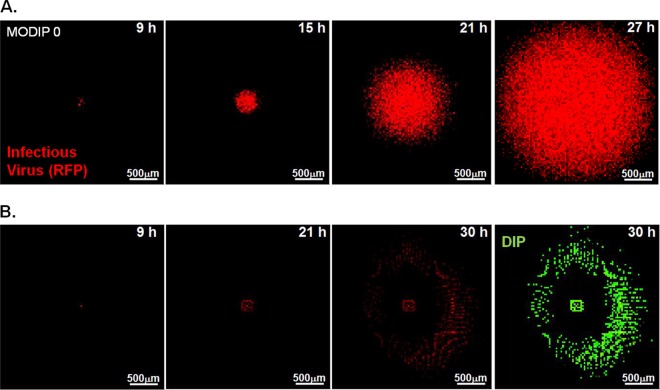
Simulated spread phenotypes using the optimum parameter set 1. The model parameters are the set optimized to match a spread rate with a high diffusion constant (*k*_ad_ = 0.8; *D* = 5 μm^2^/s; *t*_sup_ = 3 h; *k_c_* = 0). (A) A simulated plaque expansion pattern in the absence of DIPs successfully mimics the pattern observed, as shown in the plaque representing the major phenotype observed in 200 simulations. Red areas represent infectious-virus-producing (RFP-positive) cells. Bars, 500 μm. The time points are shown at the top right of each image. (B) Hollow rings emerge during 20% of the simulations in the presence of DIPs (at a MODIP of 1 or higher). The first three images, at 9, 21, and 30 h, show infectious-virus-producing cells (red), and the final image shows DIP-producing cells (green) at 30 h.

**FIG 8:**
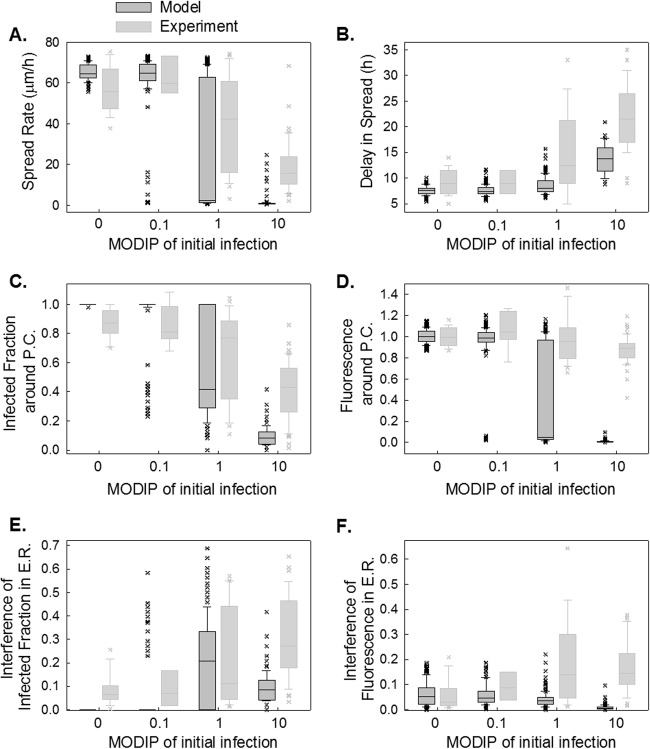
Simulated spread features using optimum parameter set 1. The spread rate (A), delay in spread (B), infected fraction (C), and fluorescence (D) around the plaque center, interference of infected fraction (E), and interference of fluorescence (F) in early rounds of spread at each MODIP (abscissa) are simulated with a model parameter set optimized for the spread rate (*k*_ad_ = 0.8; *D* = 5 μm^2^/s; *t*_sup_ = 3 h; *k_c_* = 0). Modeled distributions are shown in dark gray box plots along with the measured distributions (light gray box plots).

We cut the complexity of our CA model to four parameters (Table 2), which were adjusted to match observed features of infection spread. First, we kept the diffusion constant at 5 μm^2^/s, within the range observed previously (15, 61, 62), and fit the model to the measured spread features by optimizing the other three parameters. At the optimum model parameters (Table 2, parameter set 1), the model was able to reproduce well the spread patterns in the absence of DIPs (Fig. 7A), including the key aspects of infection spread, such as the spread rate (Fig. 8A, MODIP 0) and the decrease in fluorescence at the infection front (Fig. 8F, MODIP 0), comparably to the performance of previous spread models (15, 62). However, in the presence of DIPs, this model failed to exhibit complete inhibition of spread at any MODIP and caused gaps in the infection front, resulting in the formation of hollow rings (Fig. 7B), which emerged in >40% of the simulations at MODIP levels of 1 and 10. Despite its high frequency in simulations, the hollow-ring structure was not observed in the experiments, suggesting that this pattern may be unrealistic. Also, because of the discontinuity in the hollow-ring patterns, the spread features could not be precisely extracted.

**TABLE 2:** Parameter values of the model^*a*^

Parameter	Range	Value with:	
		Parameter set 1^*b*^	Parameter set 2^*c*^
Adsorption efficiency (*k*_ad_)	0.5–1	0.8	0.5
Diffusion constant (*D*) (μm^2^/s)	10^−4^–10^*d*^	5	0.025
Superinfection period (*t*_sup_) (h)	0.5–4^*e*^	3	1.5
Clearance rate (*k_c_*)	0–0.5	0	0.1

a Model parameters for infectious virus and DIPs were identical.

b With a high diffusion constant.

c To capture spatial patterns.

d See reference 15.

e See reference 60.

**FIG 9:**
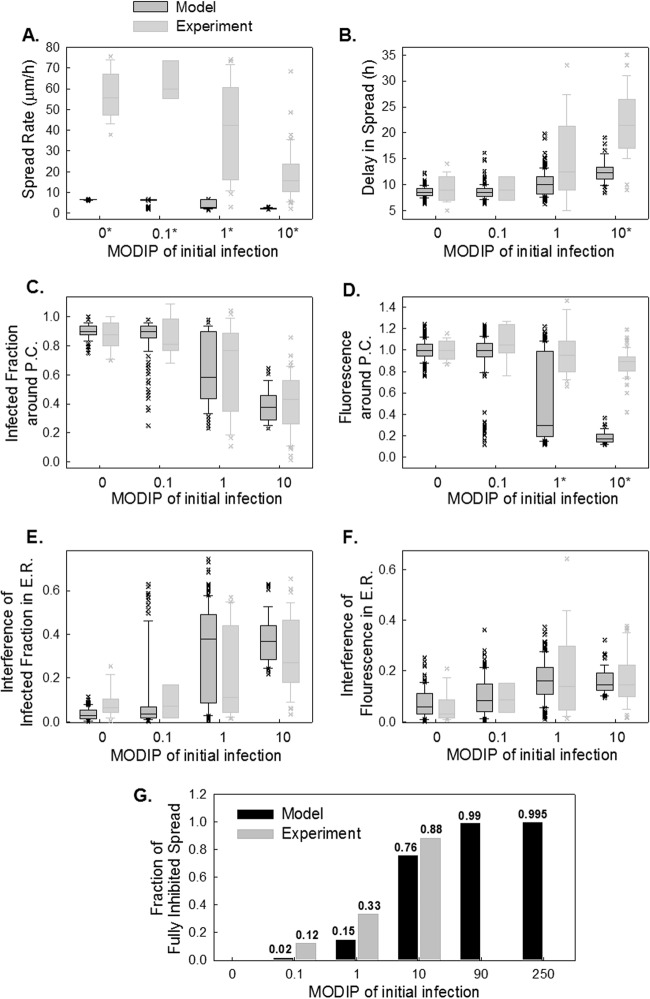
Simulated spread features obtained using optimum parameter set 2. (A to F) Spread rate (A), delay in spread (B), infected fraction (C), relative fluorescence around the plaque center (D), interference in the infected fraction (E), and interference in relative fluorescence in early rounds of spread (E.R.) (F) at each MODIP (abscissa) are simulated with a model parameter set optimized to capture spatial patterns (*k*_ad_ = 0.5; *D* = 0.025 μm^2^/s; *t*_sup_ = 1.5 h; *k_c_* = 0.1). Modeled distributions are shown in dark gray box plots along with the measured distributions (light gray box plots). A star next to a MODIP label indicates a significant difference (*P* < 0.01) between the measured and modeled distributions of the associated feature. (G) The fraction of fully inhibited spread observed in experiments (gray bars) is compared with that estimated by the model (black bars) under tested (MODIP, 0 to 10) and untested (MODIP, 90 or 250) MODIP conditions.

**FIG 10:**
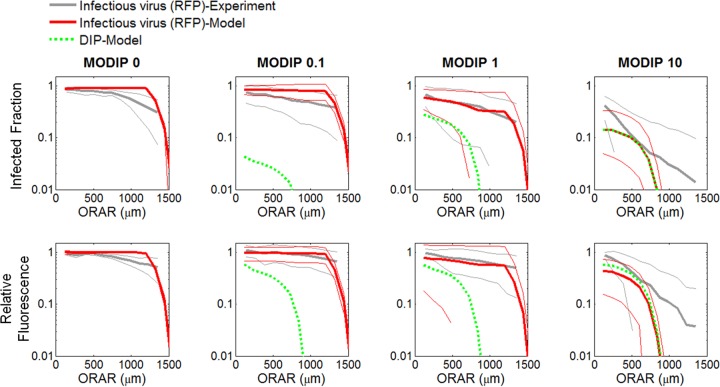
Comparison of experimental and computational (CA model) patterns of coinfection spread. The infected fraction (ordinate) (top) and relative fluorescence (ordinate) (bottom) over concentric rings around the plaque center (ORAR [abscissa]) from experiments (gray lines) and models (red lines) are shown. The thick and thin solid lines represent means and standard deviations, respectively. Green dashed lines represent the fractions of DIP-producing cells and relative DIP yields from the CA model. All CA models employed parameter set 2.

**FIG 11:**
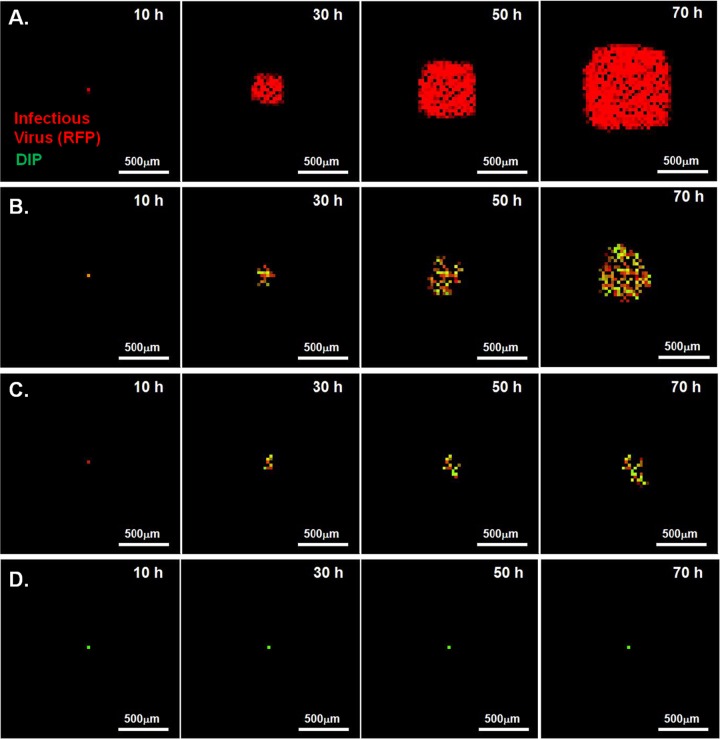
Simulated spread of virus-DIP coinfection using optimum parameter set 2. Infectious virus (RFP) and DIP yields from cells are shown in red and green, respectively. The intensity of the color indicates the extent of yield. Bars, 500 μm. Each phenotype was selected from 200 simulations to represent major spread patterns. (A) Simulated spread of a normal plaque spreading in the absence of DIPs (MODIP, 0). (B) Simulated spread of a slow-growing plaque (MODIP, 1). (C) Simulated spread of a patchy plaque (MODIP, 10). (D) Simulated fully inhibited spread. See also Movies S4 to S6 in the supplemental material. All CA models employed parameter set 2.

The mismatches between the experimental observations and the spatial spread features estimated by the model with a high diffusion constant led us to optimize the model parameters based on spatial patterns (optimum parameter set 2 in Table 2). The fit resulted in a low diffusion constant and low spread rates (Fig. 9A), but the distributions of delay in spread (Fig. 9B) and spatial features (Fig. 9C to G) were captured well. Particularly, the changes observed in the infected fraction around the plaque center (Fig. 9C), and its drop in neighboring cells (Fig. 9E and 10), as the MODIP increased matched the model results very well. Similarly, in the model, the complete inhibition of spread occurred at a frequency comparable to that in the experiments (Fig. 9G). Extrapolation of the DIP levels to an experimentally untested range using the model suggested that the probability of complete inhibition of infection spread increases further with an increasing MODIP, but the possibility of virus propagation cannot be fully prevented, even at a MODIP of 250. Moreover, the model successfully reproduced the fraction of fluorescence-positive cells and the relative fluorescence in these cells at each concentric ring centered on the initially infected cell (Fig. 9). In other words, the effects of MODIP and DIP/virus production from a cell on the progression of infection spread were well represented by the model, allowing us to reproduce the observed spread phenotypes (Fig. 11; see also Movies S4 to S6 in the supplemental material). Although the estimates of delay in spread (Fig. 9B) and relative fluorescence around the plaque center (Fig. 9C) under high MODIP conditions (1 and 10) were lower than the actual values (*P* < 0.01), considering the richness of the experimental data, the model provided a good fit to the majority of the experimental observations overall and provided a platform on which to test the effects of different aspects of virus infection and spread.

### Simulated infection spread is sensitive to parameters of extracellular processes.

To probe the sensitivity of infection spread to each model parameter, we perturbed one parameter at a time, while holding all other parameters at their optimum values (Table 2), and we scored the significance of change in the distributions of different spread features using the Mann-Whitney U test. As summarized in Table 3, increasing the adsorption efficiency and clearance rate reduced the frequency of successful infections, as reflected by the drop in reporter-positive cells and the rise in fully inhibited spread. While higher adsorption efficiencies enhanced DIP input into cells, increasing the clearance rate reduced the level of extracellular infectious virus, and both conditions reduced the probability of successful infection. Interestingly, the effects of these two model parameters on relative mean fluorescence were opposite, potentially because of the exclusion of fully inhibited spread patterns from the quantification of mean fluorescence distributions. Overall, the spread rate and the delay in spread were most sensitive to the diffusion constant. In contrast, these properties of spread, as well as the fraction of fully inhibited plaques, were insensitive to the superinfection period. Increasing the superinfection period led to a higher number of successful infections, since a longer superinfection time enables the accumulation of additional infectious virus and thus increases the chance for a successful infection.

**TABLE 3:** Effects of model parameters on simulated infection spread

Parameter	Effect^*a*^ on:			
	Adsorption efficiency (*k*_ad_)	Clearance rate (*k_c_*)	Superinfection period (*t*_sup_)	Diffusion constant (*D*)
Spread rate	↓	↓	↓	↑ for *D* of >0.1
Delay in spread	↔	↔	↔	↓
Relative fluorescence around P.C.	↑	↑	↓	↓ for *D* of <0.1, ↑ for *D* of >0.1
Infected fraction around P.C.	↓	↓	↑	↑
Fraction of fully inhibited spread	↑	↑	↔	↓ for *D* of <0.1

a ↑, increasing with an increase in the parameter (*P* < 0.01); ↓, decreasing with an increase in the parameter (*P* < 0.01); ↔, no significant change (*P* > 0.01).

## DISCUSSION

The spread of infection in a human host is a complex process, linking biology and physics. Viral gene expression, activation of innate defensive cytokine production, and stimulation of cell-mediated adaptive immune responses combine with physical movement of virus particles and cytokines released from their initial infected cellular sources to naïve cells and tissues. The process becomes still more complex when defective interfering particles enter the mix, coinfecting susceptible cells, perturbing normal viral replication, triggering cellular responses and signaling, and spreading to near or distant naïve cells and tissues. Here we have taken initial steps to dissect and reconstruct parts of virus-DIP interactions within cells and the subsequent impacts on infection spread in a plaque growth system, neglecting, for now, contributions from host immunity or physical transport by processes other than free diffusion, factors that have been considered elsewhere (10, 50, 55, 56, 63–67).

In the absence of DIPs, infectious virus propagated uniformly at a constant rate (Fig. 2 and 3), as in previous studies (15, 55). Plaques initiated by a cell coinfected with a low DIP level limited viral gene expression, reducing the rate of infection spread (Fig. 2, slow growth). When a cell coinfected with a high DIP level initiated plaque growth, not only was the rate of spread reduced, but the spread was also asymmetric (Fig. 2, patchy plaque). In both cases, the inhibitory effects of DIPs on the frequency and yield of successful infections (Fig. 3C and D and 5D and E) and the spread rate (Fig. 3B and 4C) provide evidence that DIPs within the population become enriched relative to viable virus as the infection propagates. Thus, at larger radii, cells may be coinfected with higher input DIPs (MODIP) and lower infectious virus (MOI) than cells closer to the plaque center. Lower MOI correlates with greater cell-to-cell variation in virus production (18, 19, 68, 69), which may have contributed to the spatial heterogeneity in viral activity that we observed in early infected cells near the center of patchy plaques or in the later infected cells at the outer edges of slowly expanding plaques (Fig. 2; Movies S2 and S3). Moreover, the heterogeneity in viral activity was also apparent within individual plaques (standard deviations in Fig. 4 and 5), reflecting the variability of growth in the initial coinfected cells as well as in early rounds following spread to neighboring cells. Despite such heterogeneity, the correlation between patterns of infection over multiple cycles and the DIP level of the initial coinfected cell (Fig. 4 and 5) highlights a potential key sensitivity to initial conditions. Specifically, the timing and level of DIP production from the initial coinfected cell guides how subsequent rounds of infection behave. Ultimately, the sensitivity of within-host spread to initial conditions may impact the probability of infection transmission to a new susceptible host.

The patchy appearance of plaques initiated by individual cells coinfected with higher levels of DIPs suggests that the regions immediately adjacent to the patch (of viral reporter expression) contain cells that do not express enough fluorescent protein to be detected. Such a situation could arise if the cells were enriched in DIPs at a level sufficiently high to suppress amplification of the viable viral genome and its associated expression of the reporter protein. Thus, dark regions near patches may be DIP rich and virus poor. Because the initiating population of DIPs is potentially quite heterogeneous with respect to DIP species and their corresponding interfering activities (51), different DIP species might be enriched along different directions of plaque expansion, giving rise to unpredictable patch-like patterns. Moreover, if DIPs reach very high levels within cells, they can interfere with their own replication (45), thereby enabling wild-type virus to “escape” and be enriched, which might provide a mechanism for new patches to arise at positions distant from the initial coinfected cell.

The presence of DIPs expanded the variability in the range of gene expression within cells and infection spatial spread across cell populations. To probe the link between intracellular gene expression and extracellular infection spread, we used a cellular automaton (CA) model. Past CA models have tested the sensitivity of simulated spatial spread on various simulation parameters (13–16, 70). In contrast, our approach, summarized in Fig. 6, employed measured single-cell distributions of virus and DIP production to inform the amplification and subsequent spatial spread of virus-DIP coinfections. The model successfully captured the DIP-induced changes in spread patterns and the variability in these patterns (Fig. 8 to 10), in agreement with a mechanism where patterns of infection spread reflect the coupling between cell-to-cell variation, prevalent in the absence of DIPs, and the interfering effects of DIPs on virus intracellular growth.

Key parameters of previous models of infection spread (13, 14) have lacked an experimental basis. In contrast, the incorporation of experimentally observed distributions based on single-cell measures has enabled our model to define four key biophysical parameters that may be estimated from independent experiments: virus diffusivity, virus adsorption on cells, superinfection period, and virus clearance, accounting for virus degradation and immobilization of virus or DIPs by the agar overlay. By using these parameters and keeping the diffusion constant high, the model successfully captured the temporal and spatial features of spread in the absence of DIPs (Fig. 7A; Fig. 8, MODIP 0), showing that the model performed as well as previous CA models of infection spread (15). However, the high diffusion constant contributed to large gaps in simulated virus propagation, apparent in the presence of DIPs as hollow rings (Fig. 7B), leading to underestimates of the interference during plaque propagation (Fig. 8E and F). These results suggest that the high diffusion constant estimated by earlier models (15) does not apply to all coinfection conditions. It is possible that spatial gaps arise owing to the oscillatory dynamics between virus and DIPs (48, 71–73); however, the large size of the gaps that arose in our simulations highlights a need for further investigation. The simplest refinement to our model was achieved by using a smaller diffusion constant (Table 2; Fig. 9 and 11). In this case, increasing the rate of diffusion enhances the virus propagation rate, while the adsorption efficiency and the superinfection period determine the extent to which the propagating virus can successfully enter a susceptible cell and influence the infection outcome at the cell level. Our model indicates that a low adsorption efficiency and a short superinfection time reduce infectious virus inputs into cells, but they also maintain a moderate number of virus particles in the extracellular virus pool, which enables the virus and DIPs to propagate further across the cell monolayer (Table 3). Otherwise, cells can serve as an adsorption sink for all particles and thereby slow the spread rate.

Moreover, all spread patterns that exhibited interference also showed the coexistence of infectious virus and DIPs in most of the cells (Fig. 10). However, even in spread patterns initiated at a high DIP level, the fraction of DIP-producing cells did not exceed that of infectious virus (RFP)-producing cells (Fig. 9), suggesting that the production and enrichment of DIPs remains limited to cells also infected with intact virus.

The model enabled the extrapolation of experimental results to untested conditions, as well as the visualization of DIP coinfections. Simulations initiated with high DIP levels showed a complete inhibition of spread and a high level of DIP generation in an initially infected cell (Fig. 9D). Specifically, our model indicates that the probability of spreading infection can be reduced to <1% for an input of at least 90 DIPs per cell. However, complete termination of infection spread was not possible, even up to a MODIP of 250 (Fig. 9G). It should be noted that no implementations of our model include DIP-mediated interference with DIP production, which enables recovery of infectious virus titers at high levels of coinfecting DIPs (45, 74). The incorporation of such mechanisms would be expected to contribute to the persistence of infection spread in the presence of DIPs.

### Conclusion.

The extent of DIP and virus particle production from individual coinfected cells depends on the input levels of DIPs and virus particles. Further, individual cells exhibit extreme heterogeneity in the production of DIPs and virus particles. These effects of the initial dose and stochastic particle production contribute to dynamic patterns of infection spatial spread, which we have characterized and elucidated by quantitative image processing and model building. More importantly, we have established a framework with which to study multiscale characteristics of virus infections that may be extended to other cell-virus systems and host environments, including viral infection dynamics *in vivo* (68, 75, 76).

## Supplementary Material

Supplemental material
