# Supplementary material for: Spatial-Temporal Patterns of Viral Amplification and Interference Initiated by a Single Infected Cell
Source: J Virol. 2016 Jul 27;90(16):7552–66. doi: 10.1128/JVI.00807-16 (PMC4984635; doi:10.1128/JVI.00807-16)
Supplement: Supplemental material [file supp_90_16_7552__index.html]

Spatial-Temporal Patterns of Viral Amplification and Interference Initiated by a Single Infected Cell — Supplemental material 

# Spatial-Temporal Patterns of Viral Amplification and Interference Initiated by a Single Infected Cell

## Supplemental material

- Supplemental file 1 -

  Legends to Movies S1–S6

  PDF, 22K
- Supplemental file 2 -

  Movie S1 (Normal plaque growth (Fig. 2).)

  AVI, 331K
- Supplemental file 3 -

  Movie S2 (Slow plaque growth (Fig. 2).)

  AVI, 102K
- Supplemental file 4 -

  Movie S3 (Patchy plaque growth (Fig. 2).)

  AVI, 51K
- Supplemental file 5 -

  Movie S4 (Simulated normal plaque growth (Fig. 11).)

  AVI, 829K
- Supplemental file 6 -

  Movie S5 (Simulated slow plaque growth (Fig. 11).)

  AVI, 625K
- Supplemental file 7 -

  Movie S6 (Simulated patchy plaque growth (Fig. 11).)

  AVI, 561K
