# Supplementary material for: Spatial-Temporal Patterns of Viral Amplification and Interference Initiated by a Single Infected Cell
Source: J Virol. 2016 Jul 27;90(16):7552–66. doi: 10.1128/JVI.00807-16 (PMC4984635; doi:10.1128/JVI.00807-16)
Supplement: Supplemental material [file JVI.00807-16_zjv999181865so1.pdf]

Legends for supplemental movies
Akpinar et al. 2016

Movie 1
normal plaque growth (Fig 2)

Movie 2
slow plaque growth (Fig 2)

Movie 3
patchy plaque growth (Fig 2)

Movie 4
simulated normal plaque growth (Fig 11)

Movie 5
simulated slow plaque growth (Fig 11)

Movie 6
simulated patchy plaque growth (Fig 11)
